# Alcohol-free essential oils containing mouthrinse efficacy on three-day supragingival plaque regrowth: a randomized crossover clinical trial

**DOI:** 10.1186/s13063-017-1901-z

**Published:** 2017-03-31

**Authors:** Enrico Marchetti, Simona Tecco, Eleonora Caterini, Fabio Casalena, Vincenzo Quinzi, Antonella Mattei, Giuseppe Marzo

**Affiliations:** 1 Department of Life, Health and Environmental Sciences, University of L’Aquila, P. le G. Liberatore, Ed. Delta 6, 67100 L’Aquila, Italy; 2 Dental School University Vita-Salute San Raffaele, Milano, via Le Mainarde 26, Pescara, 65124 Italy

**Keywords:** Dental plaque, Mouthrinse, Essential oils, Alcohol-free, Chlorhexidine

## Abstract

**Background:**

To evaluate the antiplaque effects of an alcohol-free mouthrinse containing essential oils—Listerine Zero (LZ)—and an alcohol-based essential oils mouthrinse (EO+) compared with a positive control of 0.20% chlorhexidine mouthrinse (CHX) and a negative control of a placebo solution (saline), using an in vivo plaque regrowth model of three days.

**Methods:**

The study was designed as a double-masked, randomized, crossover clinical trial, involving 21 volunteers to compare four different mouthrinses, using a three-day plaque regrowth model. After receiving thorough professional prophylaxis at baseline, over the next three days each volunteer refrained from all oral hygiene measures and performed two daily rinses with 15 mL of the test mouthrinses. EO+ was compared with LZ. CHX rinse served as a positive control and a placebo solution as a negative control. At the end of each experimental period, the Plaque Index (PI) was assessed and a panelist completed through a visual analogue scale (VAS) questionnaire evaluating the organoleptic properties of each product. Each participant underwent a 14-day washout period and then there was another allocation.

**Results:**

LZ showed the same inhibitory activity on plaque regrowth compared with EO+ in the whole mouth (PI = 1.72 versus 1.65, respectively), but there was less of an effect compared to the CHX (overall PI of 1.07) and a more efficient activity than the saline solution negative control (PI = 2.31). The difference of 0.07 between LZ and EO+ was not statistically significant.

**Conclusions:**

LZ seems to have the same inhibiting effect on plaque regrowth as EO+ and a less inhibiting effect than the CHX control. Both LZ and EO+, as well as the CHX control, show a better inhibiting effect on plaque regrowth than the placebo solution.

**Trial registration:**

ClinicalTrials.gov, NCT02894593. Registered on 4 September 2016.

**Electronic supplementary material:**

The online version of this article (doi:10.1186/s13063-017-1901-z) contains supplementary material, which is available to authorized users.

## Background

Plaque is a biofilm of microorganisms responsible for the development of caries and periodontal disease. The daily removal of supragingival dental plaque represents a major factor in the prevention of caries, gingivitis, and periodontitis [1, 2]. Plaque control is largely obtained by daily effective tooth-brushing and inter-dental cleaning; plaque removal can be improved by adding the use of a mouthrinse.

An effective action of chemical formulation (F.D.I. Commission 2002) (especially antiseptics) to control plaque and gingivitis levels has been proved [3].

Most mouthrinses contain an alcohol (especially ethanol) in order to act as a carrier agent for active essential oils to penetrate the plaque and to give the user a “clean mouth sensation” [4].

The alcohol content of mouthrinses, besides having antiseptic properties, serves the purpose of breaking down or dissolving active principles, in addition to that of preserving the formula components, although such content does not directly contribute to effective biofilm and gingivitis control [5]. However, there are some contraindications in the use of alcohol-based mouthrinses, like the use by infants, pregnant women, alcohol addicts, and patients with mucosal injuries. There are also some undesirable effects, like burning or a sore sensation, or a painful sensation for patients with existing soft tissue injuries, or a perception of dryness in the mouth [5].

In order to avoid the use of alcohol-based mouthrinses in particular conditions, scientific interest is becoming more widespread in introducing a mouthrinse with strong anti-plaque qualities and no alcoholic ingredients.

One of these product is the alcohol-free essential oils mouthrinse Listerine Zero (LZ), which has little documentation with regard to its anti-plaque effects.

### Specific objective

The aim of this study was to evaluate the antiplaque effects of an alcohol-free essential oils mouthrinse (LZ) and an alcohol-based essential oils mouthrinse (EO+) compared with a positive control of a 0.2% chlorhexidine mouthrinse (CHX) and a negative control of a placebo solution (saline), using an in vivo plaque regrowth model of three days.

## Methods

### Trial design

The study was designed as a double-masked, four-groups, randomized, controlled, crossover clinical trial.

The study was conducted in the Division of Periodontology, Dental Clinic, University of L’Aquila from January to October 2015.

The volunteers were assigned the active or control solutions in a randomized sequence. Randomization and allocation of active or control solutions were undertaken by a person not directly involved in the research project. No changes to methods after the trial beginning (such as eligibility criteria) were performed (Additional file 1).

### Participants

**Fig. 1:**
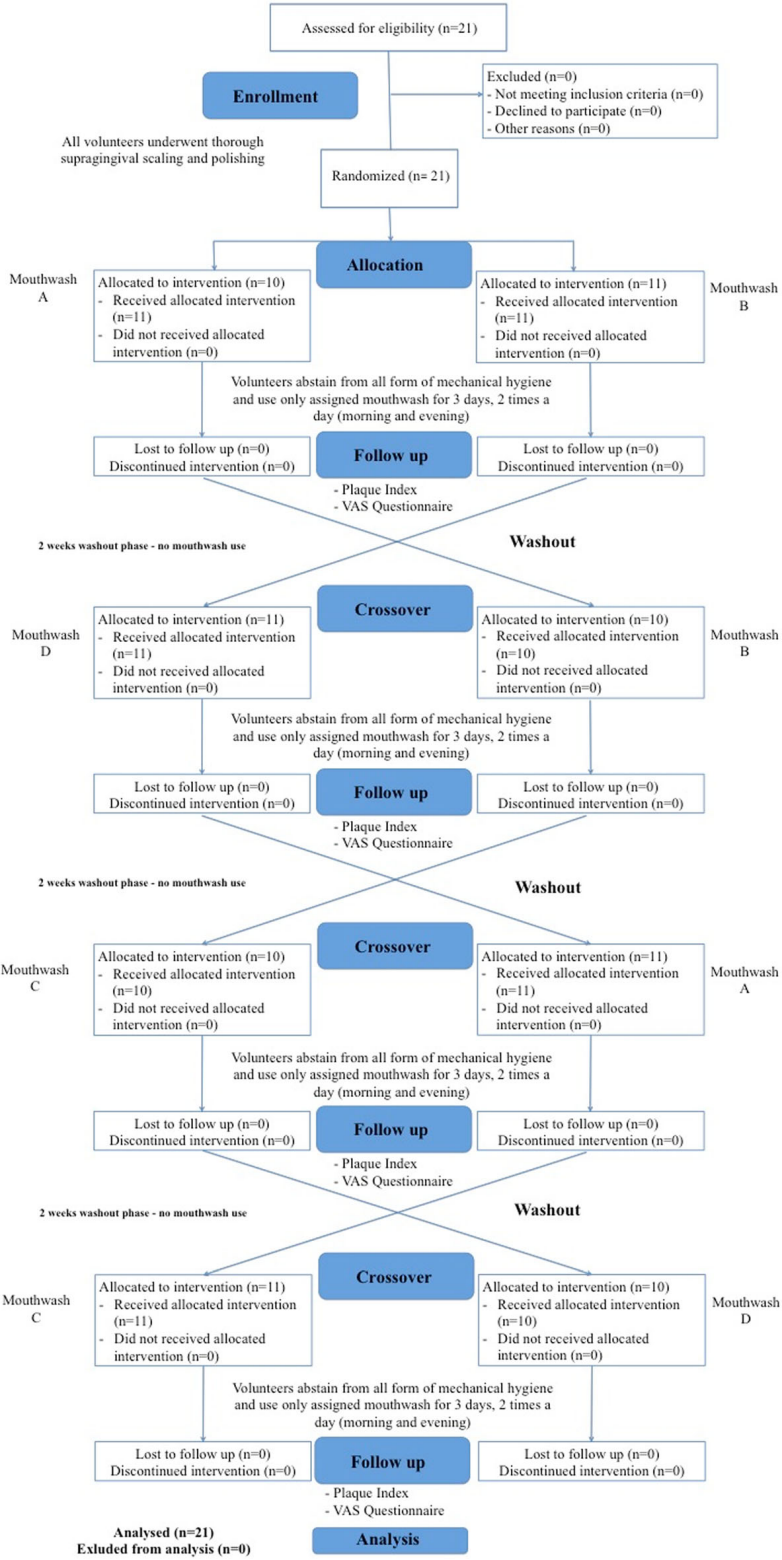
*Flow chart* of the study

Twenty-one (21) dental hygiene student volunteers (14 women, 7 men; age range 21–41 years; mean age 26.2 ± 4.3 years) participated in the study. The participants were recruited through the near-graduate student who promoted the graduation study. All of the candidates were screened for suitability by the research team. The selection inclusion criteria were: dentition with ≥ 20 evaluable teeth (minimum of five teeth per quadrant) and age of majority; exclusion criteria were oral lesions, severe periodontal problems (probing depth ≥ 5 mm or attachment loss > 2 mm), and removable prostheses or orthodontic bands/appliances. Participants allergic to several mouthrinse components were excluded from the study. All eligible volunteers were given oral and written information about the products and the purpose of the study and were asked to sign an informed consent form. The study flow chart is shown in Fig. 1.

Regarding the habits of participants, only six participants smokers (they smoked less than five cigarettes/day). All the participants declared themselves accustomed to using mouthrinses, although not assiduously. Moreover, all the participants said they had tried various types of mouthrinses in the past.

### Interventions

On day 1 of each of the four study periods, after baseline examinations consisting of an oral soft and hard tissue examination, the participants received a complete dental prophylaxis and professional scaling and polishing to remove all plaque and extrinsic tooth stains. This process was performed using hand instruments, mechanical scalers, rotating brushes with polishing paste, and dental floss in the interproximal areas. To ensure that all deposits were removed, a second polishing episode was performed after the use of a disclosing solution. The volunteers were assigned test solutions in a randomized sequence. The allocation of active or control solutions was undertaken by a person not directly involved in the research project. The participants received the bottles of mouthrinses containing LZ mouthrinse, alcohol-based EO+ mouthrinse, chlorhexidine 0.2% (CHX) mouthrinse, and a flavored placebo solution (saline) (Table 1).

**Table 1:** Products and their compositions

Group	Product and manufacturer	Composition	Instruction
LZ	Listerin Zero® formulation Johnson & Johnson Consumer Inc.	sodium fluoride 0.02% (0.01% w/v fluoride ion), water, sorbitol solutio, propylene glycol, poloxamer 407, sodium lauryl sulfate flavor, sodium benzoate phosphoric acid, eucalyptol, methyl salicylate, thymol, sodium saccharin, menthol, disodium phosphate, sucralose, FD&C Red No. 40, FD&C Blue No. 1	All of the participants were instructed to refrain from using any other means of oral hygiene during the experimental period. All of the participants were instructed to rinse twice per day, in the morning and in the evening, with 15 mL of solution for 60 s. Subsequent rinsing with water was not allowed.
EO+	Listerine Difesa Denti e Gengive® formulation Johnson & Johnson Consumer Inc.	eucalyptol 0.092%; menthol 0.042%; methyl salicylate 0.060%; thymol 0.064%. water, alcohol (21.3%), sorbitol, benzoic acid, poloxamer 407, sodium benzoate, sodium saccharin, sodium fluoride, CI 47005, CI 42053.	
CHX	Meridol Clorexidina 0.2% Gaba International AG	Aqua, glycerin, sorbitol, PEG-40 hydrogenated castor oil, clorhexidine digluconate, aroma, citric acid, CI 42051.	
Saline		Hydro non-alcoholic solution flavored with thyme oil (8 drops per liter of water)	

New identical and anonymous bottles were used for each administration. All of the bottles containing mouthrinse were pre-weighed. All of the participants were instructed to refrain from using any other means of oral hygiene during the experimental period; to rinse twice per day, in the morning and in the evening, with 15 mL of solution for 60 s, as their sole oral hygiene measure. Subsequent rinsing with water was not allowed. Written instructions were provided explaining how to use the mouthrinse. Rinsing was performed at home without supervision. To check for compliance, the participants were asked to note the times of day when they rinsed.

After three days, all of the volunteers were examined with an erythrosine solution and the plaque in both groups was recorded at six sites per tooth using the Quigley and Hein index [6], as modified by Turesky et al. [7] and further modified by Lobene et al. [8].

All of the measurements were obtained under the same conditions by the same blinded investigator (EC). The examiner was trained and calibrated in the plaque scoring system. All of the returned mouthrinse bottles were weighed to calculate the amount of mouthrinse used and to check for compliance.

All of the volunteers then received a questionnaire that used a visual analog scale (VAS), designed to evaluate their attitudes toward the products used and especially the organoleptic properties of the mouthrinses. The patients had to report about the flavor of the mouthrinse, the permanence of the sensation after the rinse, and the eventual alteration in the taste and smell of food and drinks. They also had to give their personal impressions on the anti-plaque properties of the mouthrinse they used. The participants marked a point on a 10-cm-long, uncalibrated line with the negative extreme response (0) at the left end, and the positive extreme response (10) at the right end [9–11].

A washout period of 14 days was instituted between the treatments [9], during which the participants resumed their normal oral hygiene habits; following this two-week washout period, all of the participants again underwent a session of scaling to obtain a Plaque Index (PI) of 0 and the procedures were repeated with participants using another rinse.

### Data analysis

The plaque score was used as the main response variable. Data analysis was performed on individual PI means ± standard deviations calculated at the subject level and considered both the totality of the oral cavity, and the upper and lower anterior (incisors and canines) and posterior (premolars and molars) sites. Data were analyzed for normality of distribution with the Shapiro–Wilk test. Because the data did not result in a normal distribution, a non-parametric analysis of variance (Friedman test) was performed to determine differences among the tested products (total difference). In the presence of significant differences, post-hoc analysis for comparison of pairs of treatments were made with Wilcoxon signed-rank test with Bonferroni adjustment for multiple comparisons, and thus significance for the univariate analyses was assessed at *p* < 0.0125. Data considering the VAS scores of the questionnaire were also analyzed using the same tests. For all other analyses, to the exclusion therefore of post-hoc analysis, a 5% significance level was adopted, and the data were analyzed using the Stata/IC 12.1 statistical package.

## Results

Regarding periodontal status, pre-existing periodontal health was generally good in the whole sample. No signs of gingivitis were present in the whole sample.

All of the participants (n = 21) completed the experimental period and there were no missing values. The returns of each product suggested acceptable compliance with the instructions. No adverse events or side effects were reported.

The plaque scores for each solution at the end of the experimental period are shown in Table 2. Table 3 reports post-hoc analyses.

**Table 2:** PI scores for each treatment (mean ± SD; n = 21)

	Treatment/groups				*p* value^a^ Total differences
	CHX	EO+	LZ	Saline	
Overall	1.07 ± 0.20	1.65 ± 0.35	1.72 ± 0.36	2.31 ± 0.42	<0.0001
Vestibular + oral	1.07 ± 0.20	1.67 ± 0.35	1.73 ± 0.36	2.32 ± 0.42	< 0.0001
All vestibular	1.29 ± 0.35	1.99 ± 0.46	2.09 ± 0.43	2.85 ± 0.61	< 0.0001
Oral	0.85 ± 0.24	1.34 ± 0.31	1.37 ± 0.38	1.78 ± 0.36	< 0.0001
Upper arch	1.08 ± 0.28	1.64 ± 0.37	1.77 ± 0.41	2.47 ± 0.44	< 0.0001
Lower arch	1.05 ± 0.24	1.69 ± 0.44	1.69 ± 0.36	2.17 ± 0.46	< 0.0001
Molar	1.16 ± 0.29	2.18 ± 0.48	2.15 ± 0.43	2.54 ± 0.46	< 0.0001
Premolars	0.97 ± 0.26	1.54 ± 0.40	1.67 ± 0.49	2.24 ± 0.38	< 0.0001
Canines	1.05 ± 0.30	1.45 ± 0.43	1.57 ± 0.36	2.30 ± 0.53	< 0.0001
Incisors	1.07 ± 0.41	1.40 ± 0.39	1.47 ± 0.47	2.17 ± 0.56	0.0001

^a^Using Friedman test

**Table 3:** Post-hoc analysis

	*p* value^a^	*p* value^a^	*p* value^a^	*p* value^a^
	CHX vs. EO+	EO+ vs. LZ	LZ vs. Saline	CHX vs. LZ
Overall	0.0001	n.s	0.0002	0.0002
Vestibular + oral	0.0001	0.0001	0.0002	0.0001
All vestibular	0.0002	n.s	0.0002	0.0002
Oral	0.0001	n.s	0.0028	0.0001
Upper arch	0.0001	n.s	0.0002	0.0002
Lower arch	0.0001	n.s	0.0015	0.0002
Molar	0.0001	n.s	0.0124	0.0001
Premolars	0.0002	n.s	0.0005	0.0002
Canines	0.0006	n.s	0.0001	0.0010
Incisors	0.0100	n.s	0.0006	0.0072

^a^Using Wilcoxon signed-rank test with Bonferroni adjustment for multiple comparison, *p* < 0.0125

*n.s.* no statistically significant difference between each treatment, *p* > 0.0125

Statistical analysis showed that there were significant differences in the PI among the four groups (Tables 2 and 3).

No statistically significant differences were observed between LZ and EO+ products, in all the analyzed sites (except for the vestibular and oral site) (Table 3).

The positive control (CHX) has shown the higher inhibitory effect on plaque regrowth compared with the LZ and EO+ products; in fact, the mean overall PI was 1.07 with CHX compared with 1.65 for the EO+ product and 1.72 for the LZ product. The differences between LZ and CHX and between EO+ and CHX were all statistically significant (*p* < 0.0125).

The placebo showed less of an effect compared with the CHX, EO+, and LZ products, with an overall PI of 2.31.

The participants completed the questionnaire after each experimental period and the results are shown in Table 4.

**Table 4:** Questionnaire responses (mean and SD) determined by VAS, n = 21

							Post-hoc analysis (*p* value^a^)			
Questionnaire questions	Answers from (0) to (10)	CHX	EO+	LZ	Saline	*p* value^b^ Total difference	CHX vs. EO+	EO+ vs. LZ	LZ vs. Saline	CHX vs. LZ
1) How was the taste of the product?	Very bad Very good	4.95 ± 3.52	5.65 ± 2.70	7.5 ± 1.43	3.60 ± 2.48	0.0012	n.s	0.0116	0.0002	0.0069
2) How long did the taste remain in the mouth after rinsing?	Very long Very short	3.20 ± 3.19	4.95 ± 2.65	5.10 ± 2.25	6.70 ± 2.94	0.0114	n.s	n.s	n.s	0.0413
3) How was your taste of food and drink affected?	Negative change Positive change	3.80 ± 2.40	4.70 ± 1.90	5.20 ± 1.96	4.50 ± 1.36	n.s.	n.s.	n.s.	n.s.	n.s.
4) Was the use of the mouth rinse convenient?	Not convenient Very convenient	6.75 ± 2.84	6.20 ± 2.28	6.75 ± 2.29	3.20 ± 3.43	0.0016	n.s	n.s	0.0003	n.s
5) What is your opinion about the rinsing time?	Very long Very short	5.15 ± 1.46	2.90 ± 2.83	4.80 ± 1.91	4.95 ± 2.14	n.s.	n.s.	n.s.	n.s.	n.s.
6) What was your perception of the plaque reduction?	Insufficient Very efficient	6.25 ± 2.67	6.30 ± 2.20	6.75 ± 1.74	3.20 ± 3.47	0.0030	n.s.	n.s.	0.0011	n.s.

^a^Using Wilcoxon signed-rank test with Bonferroni adjustment for multiple comparison, *p* < 0.0125

^b^Using Friedman test, *p* < 0.05

*n.s.* no statistically significant difference between each treatment, *p* > 0.0125

With regard to the participants’ taste perception (question 1), the results demonstrated statistically significant differences with LZ product showing the best taste respect to the other products (7.5 for LZ product).

With regard to the participants’ alterations in taste perception (question 3), no statistically significant differences were noted among the products.

About the sensation of plaque reduction, LZ product was much more than the other products, with significant differences (LZ: 6.75; EO+: 6.3; CHX: 6.25; Saline:3.2; *p* < 0.0125).

## Discussion

The purpose of this study was to test the effects of an alcohol-free essential oils mouthrinse (LZ) and an alcohol-based essential oils mouthrinse (EO+) compared with a positive control (a commercially available CHX-containing product) and a negative control (a flavored saline solution as a placebo), using an in vivo plaque growth model of three days. The results confirmed the null hypothesis that there is no significant difference between LZ and the alcohol-based mouthrinses, while there is a modest but statistically significant difference in favor of the CHX product.

CHX is used as positive control because of its demonstrated ability to reduce plaque at 0.2% [12–14].

Data collection was performed using the PI (the Quigley and Hein Index, as modified by Turesky et al. and further modified by Lobene et al.), rather than the Full-Mouth Plaque Score (FMPS%), which has been employed in other studies [15] and represents the percentage of teeth surfaces with plaque accumulation. The choice of this analytical index is due to the desire to identify whether there are particular topographical sites in the mouth of the patients characterized by different effects of the three different products, as proposed in another study [11].

The PI was thus evaluated at nine different sites (global, oral area, buccal area, upper jaw area, lower jaw area, molar area, bicuspid area, canine area, and incisive area). The analysis of nine different intra-oral sites resulted in the conclusion that there are no substantial differences in the effects of the LZ and EO+ products in different areas of the mouth; this finding suggests that both mouthrinses have acceptable ability to be effective in all of the tested areas of the mouth and have substantially the same effects on all of these areas. In this study, plaque formation is evaluated only macroscopically, using the PI and not microscopically (through the bacterial count or the presence of specific periopathogens), in order to point out a clinical parameter that can be easily monitored by professionals, to control the home oral hygiene habits of patients.

All the tested products were safe and well tolerated by the patients, although the early follow-up limited the assessment of side effects, as previously hypothesized [16].

The efficacy of LZ in reducing plaque and gingivitis when compared to a placebo (a 5% hydro-alcohol mouthrinse), was first evidenced by Charles et al. [17] in a single-center, randomized, examiner-blind, two-week, no oral hygiene, parallel group, controlled clinical trial on 90 participants. LZ was more effective (*p* < 0.001) than the negative control in reducing plaque (whole mouth mean PI) and gingivitis (whole mouth mean Modified Gingival Index), and in reducing gingival bleedings for the secondary efficacy endpoint (Gingival Bleeding Index).

Cortelli et al. [18] also tested a LZ mouthrinse compared with a placebo (flavored and colored 5% hydro-alcohol mouthrinse) and with an alcohol-free cetylpyridinium chloride (CPC) containing mouthrinse, in a six-month, examiner-blind, randomized, parallel group, controlled clinical trial. The results on the 311 participants who completed the study showed that LZ had a superior efficacy in reducing plaque (whole mouth mean PI) and gingivitis (whole mouth mean Modified Gingival Index) compared with both negative control and alcohol-free CPC containing mouthrinse. However, in that study, the efficacy of LZ was not compared with an alcohol-based essential oils mouthrinse. The participants were randomized just to one of the two treatment groups and no liking ratings questionnaire followed the test.

Our trial is the first to compare LZ with an alcohol-based mouthrinse, a CHX mouthrinse, and a placebo control, and to test each of the four solutions on the whole group of volunteers after a two-week washout period and a session of scaling (to obtain a PI of 0) before the use of each solution. The aim of the current study was to evaluate the plaque regrowth index not only over the mouth, but in different sites: global vestibular, oral, upper arch, lower arch, molars, premolars, canines, and incisors. Our study also showed a liking rating scale determined by VAS respect to taste perception, duration of taste, alteration in taste perception, and convenience of use.

The pilot and feasibility study by Chalhoub et al. [19] was concerned about the effectiveness of an alcohol-free essential oils mouthwash (LZ) in institutionalized elders receiving long-term care. However, their study protocol encountered several problems, both in recruitment and in execution of the protocol; only 18 participants of the initial 25 completed the study. They were divided in two groups: a test group rinsed with 15 mL alcohol-free essential oils mouthwash twice a day for 30 s and a control group rinsed with 15 mL of tap water twice a day for 30 s. PI, denture cleanliness, and salivary levels were measured at days 0, 22, and 45. The conclusion was that the use of an alcohol-free essential oils mouthwash is not more effective than the tap water in institutionalized elders receiving long-term care. Nevertheless, the small sample size of the participants, the several limitations of the study, and the problems encountered during the execution show that larger studies about the use of an alcohol-free essential oils mouthwash in institutionalized seniors are needed.

Ulkur et al. [20], evaluating bacteria counts, show that after using an alcohol-free essential oils mouthwash for four days, the *Streptococcus mutans* colony counts are slightly higher compared with alcohol-based essential oils and alcohol-free 0.1% CHX mouthrinse, while no difference appears on the tongue surface.

They conducted a double-blind study to compare an alcohol-free 0.1% CHX mouthrinse, an alcohol-based essential oils mouthrinse, an alcohol-free essential oils mouthrinse, and a negative control in order to evaluate their effects on the *S. mutans* colonies reduction, on the teeth and tongue surfaces. All the patients had brackets on both the upper and lower dental arches. The patients were instructed to avoid any mechanical cleaning with toothbrushes or toothpicks for four days. The study collected samples at day 0, after a professional cleaning, and after a four-day plaque regrowth period, when between-group differences were detected.

As it appears, the method of Ulkur’s study contemplates different quantities, duration, and times-a-day rinse directions for each of the tested solutions, conditions that might have had effects on the resulting counts. Moreover, each of the volunteer groups tested just one type of mouthrinse, so that personal characteristics of the patients, such as salivary buffer capacity, pH, or flow rate, could have had an effect on the results.

Traditional essential oils with alcohol have been used for years as adjuncts to brushing in addressing oral hygiene, so their effectiveness in controlling plaque and gingivitis is well documented in the literature [21–26]; they appeared equivalent to CHX for long-term control of gingival inflammation, but CHX appears to perform better than alcohol based essential oils mouthrinses in plaque control [27].

However, little is known about the new formulations of essential oils mouthrinse without alcohol (alcohol-free EO), such as LZ, used in this study [9, 10].

Despite the early follow-up, patients clearly judged the tastes of the products, claiming to prefer, in a statistically significant manner, the flavors of the LZ product to the flavor of the other products. LZ was also judged as equally effective in reducing plaque respect to CHX, which does not correspond to what was found with the PI.

## Conclusions

The lowest values for the PI were obtained with CHX. LZ showed the same effect on plaque regrowth as the alcohol-based EO+ mouthrinse. Due to the short follow-up, these results could be considered preliminary and we cannot exclude that the tested products could have other effects over the medium or long term.

## Additional file

Additional file 1: Completed CONSORT checklist. (DOC 218 kb)

## Abbreviations

CHX: Positive control of 0.20% chlorhexidine mouthrinse
EO+: Alcohol-based essential oils mouthrinse
LZ: Listerine Zero
PI: Plaque Index
